# Latent inter-organ mechanism of idiopathic pulmonary fibrosis unveiled by a generative computational approach

**DOI:** 10.1038/s41598-023-49281-0

**Published:** 2023-12-11

**Authors:** Satoshi Kozawa, Kengo Tejima, Shunki Takagi, Masataka Kuroda, Mari Nogami-Itoh, Hideya Kitamura, Takashi Niwa, Takashi Ogura, Yayoi Natsume-Kitatani, Thomas N. Sato

**Affiliations:** 1 Karydo TherapeutiX, Inc., 2-2-2 Hikaridai, Seika-Cho, Soraku-Gun, Kyoto, 619-0288 Japan; 2 The Thomas N. Sato BioMEC-X Laboratories, Advanced Telecommunications Research Institute International (ATR), Kyoto, Japan; 3 National Institutes of Biomedical Innovation, Health and Nutrition, Osaka, Japan; 4 Mitsubishi Tanabe Pharma Corporation, Kanagawa, Japan; 5 Kanagawa Cardiovascular and Respiratory Center, Kanagawa, Japan; 6 Institute of Advanced Medical Sciences, Tokushima University, Tokushima, Japan; 7 V-iCliniX Laboratory, Nara Medical University, Nara, Japan

**Keywords:** Computational biology and bioinformatics, Biomarkers, Diseases, Pathogenesis

## Abstract

Idiopathic pulmonary fibrosis (IPF) is a chronic and progressive disease characterized by complex lung pathogenesis affecting approximately three million people worldwide. While the molecular and cellular details of the IPF mechanism is emerging, our current understanding is centered around the lung itself. On the other hand, many human diseases are the products of complex multi-organ interactions. Hence, we postulate that a dysfunctional crosstalk of the lung with other organs plays a causative role in the onset, progression and/or complications of IPF. In this study, we employed a generative computational approach to identify such inter-organ mechanism of IPF. This approach found unexpected molecular relatedness of IPF to neoplasm, diabetes, Alzheimer’s disease, obesity, atherosclerosis, and arteriosclerosis. Furthermore, as a potential mechanism underlying this relatedness, we uncovered a putative molecular crosstalk system across the lung and the liver. In this inter-organ system, a secreted protein, kininogen 1, from hepatocytes in the liver interacts with its receptor, bradykinin receptor B1 in the lung. This ligand–receptor interaction across the liver and the lung leads to the activation of calmodulin pathways in the lung, leading to the activation of interleukin 6 and phosphoenolpyruvate carboxykinase 1 pathway across these organs. Importantly, we retrospectively identified several pre-clinical and clinical evidence supporting this inter-organ mechanism of IPF. In conclusion, such feedforward and feedback loop system across the lung and the liver provides a unique opportunity for the development of the treatment and/or diagnosis of IPF. Furthermore, the result illustrates a generative computational framework for machine-mediated synthesis of mechanisms that facilitates and complements the traditional experimental approaches in biomedical sciences.

## Introduction

Idiopathic pulmonary fibrosis (IPF) is a chronic disease characterized by scarring in the interstitium of the lung, affecting 3–9 and 4 or less per 100,000 person-years in North America/Europe and South America/East-Asia, respectively^1,2^. Both the incidence and poor prognosis of IPF increase with age^3,4^. Specifically, the median age of the newly diagnosed is 62 years-old and their prognosis is poor—3–5 years of survival rate.

There are two Food and Drug Administration (FDA)-approved drugs for the treatment of IPF: nintedanib and pirfenidone^1,2^. Nintedanib is a tyrosine kinase inhibitor. Pirfenidone is an inhibitor of transforming growth factor (TGF)-beta production and downstream signaling, collagen synthesis and fibroblast proliferation. Hence, these drugs are regarded as pleiotropic anti-fibrosis drugs. Currently there are no IPF-specific therapeutics. Furthermore, the precise IPF diagnosis requires complex and multiple-types of tests as its overlapping pathologies with other interstitial lung fibrosis diseases^1,2^. These are in part due to the complexity of the IPF pathogenesis and to its ill-defined cellular and molecular mechanisms.

While IPF was classically considered an inflammatory disease, a new picture is emerging^1–6^. The increasing molecular and cellular evidence suggests IPF is driven by an activation of the lung epithelium. In this model, the ectopic activation of the alveolar epithelial cells results in the production of chemokines, growth factors, and extracellular matrix proteins, promoting the migration, growth, and/or differentiation of fibroblasts, and macrophages and other immune cells. Furthermore, various life-style and environmental factors, and also genetic factors are reported to influence the onset, progression, and/or mortality.

While the clinical translation of these recent advancements in understanding the IPF mechanism within the lung tissue is expected, the onset and progression of human diseases involve multiple organs -i.e., the inter-organ mechanism^7–11^. The immune responses occur in a variety of diseases such as metabolic, neoplastic, cardiovascular diseases and also in aging^12–14^. The causative roles of gut microbiota are becoming recognized in an increasing number of diseases^15^. The nervous system influences metabolic states and vice versa^16^. Metabolic dysregulation is a risk factor for many diseases and they also accelerate aging influencing the longevity^17^. Exosomes are another type of systemic factors that are associated with many types of diseases^18^. The interactions of immune cells and lung cells are involved in the pathogenesis of IPF^1,2,4–6^. Furthermore, life-style and aging are critical influencers of IPF^1,5^. Hence, it is conceivable that the inter-organ crosstalk mediated by the immune cells, systemic factors, and/or neural system could be involved in the onset, progression, and/or complications of IPF. However, very little is studied on these possibilities.

Based on this background, we postulate that the cross-talk between the lung and the non-lung organs is a part of the mechanism in the onset and/or progression of IPF. The obvious choice of the approach to test this hypothesis is to examine molecular and cellular changes in non-lung organs that accompany, precede, or follow the pathological changes of the lung in IPF. However, this approach would be difficult as the availability of non-lung tissues from the IPF patients is limited, if any.

The availability of multi-modal omics data of multiple organs and diseases is growing in the public space^19–27^. Such data space, together with computational methods, could allow us to deduce what occurs in the non-lung organs of IPF-patients and to simulate how they are regulated.

Hence, we reasoned that such a large set of multi-modal omics data across many organs and diseases in the human body provides an uncharted biomedical space where an organ-to-organ interaction that causes and/or exacerbates IPF is embedded. To uncover such a latent inter-organ mechanism of IPF, we employ a generative computational approach (also referred to as “generative artificial intelligence (AI)”)—a computational method that can produce various types of contents such as sentences, images, molecular structures, working-hypotheses(models), etc.^28^.

Towards this goal, we designed a generative computational approach as follows:

1. To detect mechanistic relatedness of IPF to non-respiratory/non-pulmonary diseases.
2. To identify molecular features that characterize the relatedness detected in 1.
3. To identify ligand–receptor relationships across multiple organs that are linked to the features identified in 2.
4. To generate a map of the inter-organ mechanism of IPF with molecular and cellular resolution that explains the findings of 1–3.

## Results

### Detection of molecular relatedness of IPF to non-respiratory/non-pulmonary diseases

**Figure 1:**
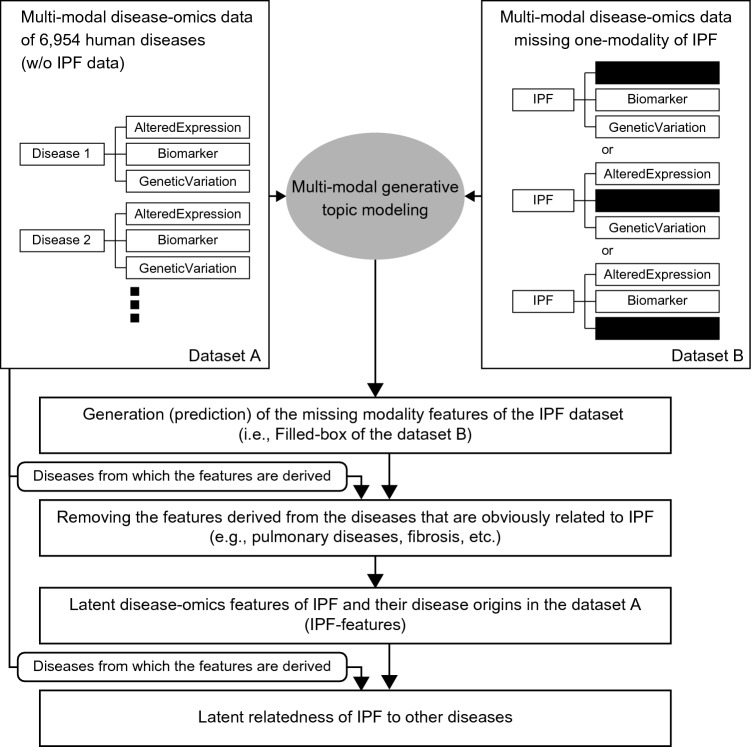
General overview of the multi-modal generative topic modeling approach for IPF. The previously developed method^29^ is adapted to IPF.

The molecular relatedness of IPF to non-respiratory/non-pulmonary diseases were identified by using the multi-modal generative topic modeling method that we developed and previously reported^29^. The overall design is summarized in Fig. 1, and it works as follows:

Two datasets are used for the multi-modal generative topic modeling: Datasets A and B. Dataset A consists of 6,954 human diseases excluding IPF, each of which is characterized by three disease omics modalities, AlteredExpression (Ae), Biomarker (Bm), and GeneticVariation (Gv), derived from DisGeNET v7.0^19,27^. “Ae” is the list of genes and proteins of which changes in expressions are associated with a corresponding disease(s). “Bm” is the list of biomarkers which are described for a corresponding disease(s). “Gv” is the list of genes of which mutations are reported for a corresponding disease(s). Dataset B consists of three types of IPF modality combination, each consisting of Bm/Gv (i.e., missing Ae), Gv/Ae (i.e., missing Bm), or Ae/Bm (i.e., missing Gv), also from DisGeNET v7.0^19,27^. The multi-modal generative topic modeling generates (i.e., predicts) the features of the missing modalities. The performance was evaluated by calculating the area under the receiver operating characteristic curve (AUC) values as previously described^29^ and they were found to be above 0.8 for all three modalities (Supplementary Fig. S1). Next, from these computationally generated features, those derived from the modalities of IPF itself and those of obviously IPF-related diseases are removed. The diseases that are obviously related are those of which names contain “Pulmonary”, “Lung”, “Fibrosis”, “Respir^**^”, “Chest”, “Pneumo**” (**could be any characters). The remaining features are now designated as “latent disease-omics features of IPF (also referred to as IPF-features)”. Moreover, IPF and the diseases from which these IPF-features are derived in Dataset A establish “latent relatedness of IPF to other diseases”.

**Figure 2:**
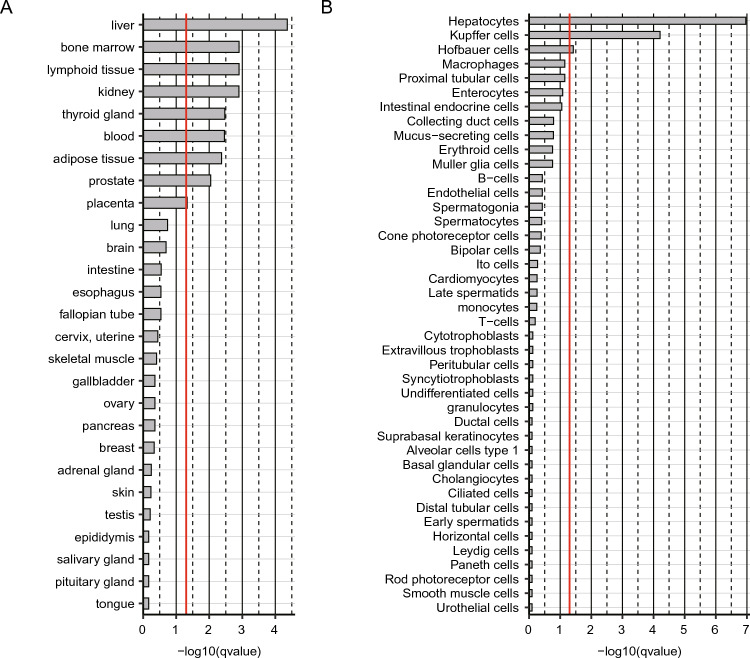
The organ and cell-enrichment analyses of the latent IPF-features. (**A**) The organ enrichment. (**B**) The cell-type enrichment. The enrichment level of the 83 IPF-features in each organ and each cell-type is shown as bar-graph of −log10(q-values) in the descending order. The q-value (qvalue) = 0.05 (the threshold for the statistical significance) is indicated as a red line in each graph. The raw data are available as Supplementary Table S2.

Using this approach, we identified 83 latent IPF-features (Supplementary Table S1). The human-organ-expression analysis using THE HUMAN PROTEIN ATLAS v 21.1^23–25^ (see also “Methods” section) found that their expression is most enriched in the liver (Fig. 2A, Supplementary Table S2). Additionally, we also detected the statistically significant (i.e., q-values $<0.05$) enrichments in the immune system (bone marrow, lymphoid tissue, blood), the kidney, the thyroid gland, adipose tissue, the prostate, and the placenta. The cellular level analysis found the highest enrichment in the hepatocytes (Fig. 2B, Supplementary Table S2). In addition, we also detected the statistically significant (i.e., q-values $<0.05$) enrichments in Kupffer cells and Hofbauer cells, two types of macrophages found in the liver and the placenta, respectively. These results suggest a possibility that the liver, in particular, hepatocytes and the intra-hepatic immune cells such as Kupffer cells, participate in the pathogenesis of IPF.

### Latent relatedness of IPF to other diseases

**Figure 3:**
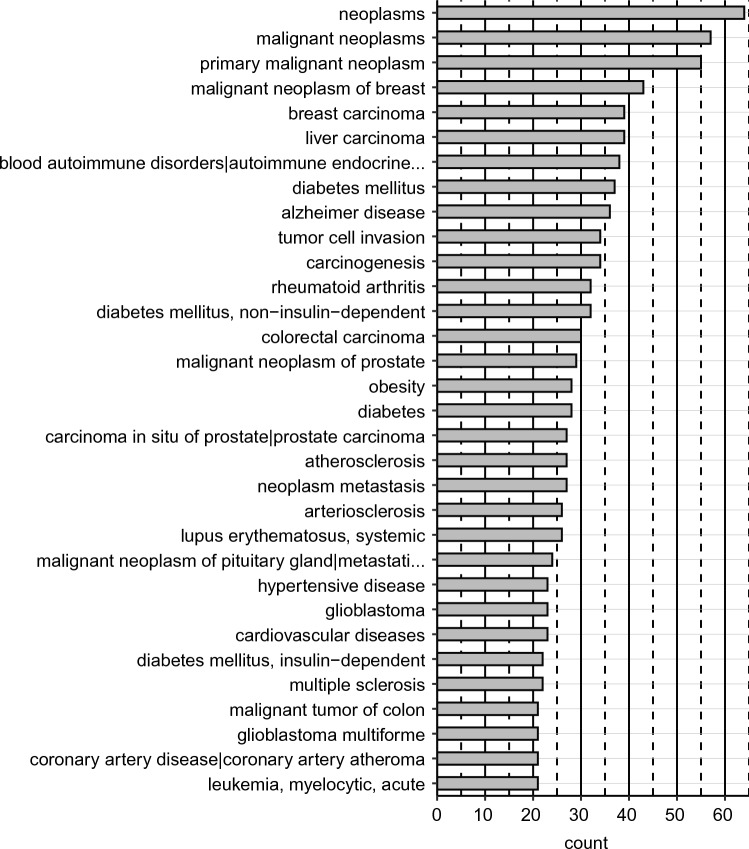
The latent diseases to which IPF is molecularly related. The frequency of the appearance of the 83 IPF-features in each disease is indicated as “count”. Shown are the diseases of which counts are above 20 in the descending order. The long disease names are cut short and indicated as “...” at their ends. The raw data are available as Supplementary Table S3.

Next, we determined the disease-label(s) of the 83 latent IPF-features to identify non-pulmonary/non-respiratory diseases to which IPF is related (Fig. 3, Supplementary Table S3) (see also “Methods” section). This analysis found these IPF-features are derived from neoplastic diseases. In addition, they are also labeled with autoimmune disorders, diabetes, Alzheimer’s disease, rheumatoid arthritis, obesity, cardiovascular diseases (atherosclerosis, arteriosclerosis, hypertensive disease, etc.), systemic lupus erythematosus, and multiple sclerosis. The result suggests that these non-pulmonary/non-respiratory diseases are related to IPF at the molecular level.

### Inter-organ mechanism of IPF

**Figure 4:**
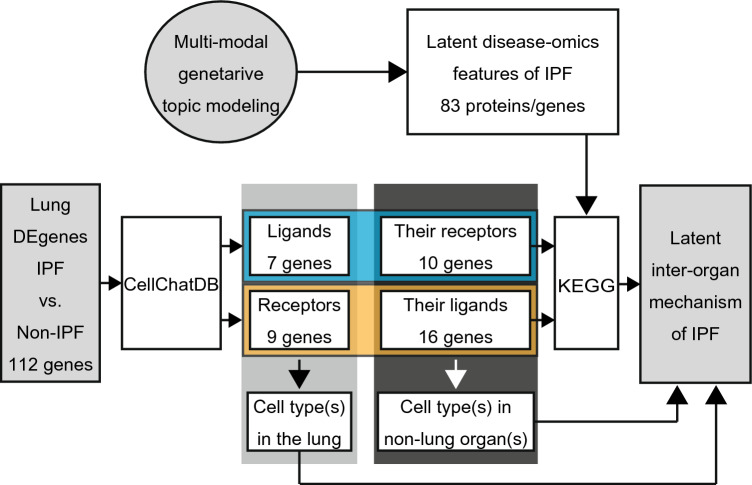
General overview of the computational framework to generate an inter-organ mechanism of IPF. See the “Methods” section for the detailed step-by-step description. The 83 latent IPF-features and 112 lung DEgenes (IPF vs. non-IPF) are found in Supplementary Tables S1 and S4, respectively.

A putative inter-organ mechanism was computationally generated as described in Fig. 4. The step-by-step description (Steps 1–7) and the results from each step are as follows:

Step 1: To identify the ligands in the lung differentially expressed genes (DEgenes) (via CellChatDB, a human ligand–receptor combination database, as described in Fig. 4).

In IPF treatments, distinguishing IPF from the other non-IPF lung diseases is most critical for the better outcome^1–6^. Therefore, we analyzed DEgenes between IPF and non-IPF lung diseases subjects, rather than those between IPF and healthy subjects.

The DESeq2 analysis of the lung tissues obtained from 95 IPF and 204 non-IPF (unclassifiable interstitial pneumonia: UCIP, idiopathic nonspecific interstitial pneumonia: NSIP, idiopathic pleuroparenchymal fibroelastosis: PPFE, other idiopathic interstitial pneumonias: IIPs, hypersensitivity pneumonitis: HP, connective tissue diseases: CTD, and other interstitial lung disease) lung disease patients (see also “Methods” section) identified a total of 112 IPF-DEgenes (Supplementary Table S4).

The CellChatDB analysis identified seven ligands (C–C motif chemokine ligand 18: CCL18, C–X–C motif chemokine ligand 9: CXCL9, C–X–C motif chemokine ligand 10: CXCL10, C–X–C motif chemokine ligand 11: CXCL11, interleukin 6: IL6, interferon gamma: IFNG, selectin E: SELE) in the 112 IPF-DEgenes (Table 1).

**Table 1:** The ligands encoded by the IPF-DEgenes and their receptors.

Ligand (IPF-DEgene)	Receptor	Evidence
CCL18	ACKR1	PMID: 26740381
CXCL9	ACKR1	PMID: 26740381
CXCL10	ACKR1	PMID: 26740381
CXCL11	ACKR1	PMID: 26740381
CXCL9	CXCR3	KEGG: hsa04060
CXCL10	CXCR3	KEGG: hsa04060
CXCL11	CXCR3	KEGG: hsa04060
CXCL11	ACKR3	KEGG: hsa04060
IL6	IL6R / IL6ST	KEGG: hsa04060
IFNG	IFNGR1 / IFNGR2	KEGG: hsa04060
SELE	CEACAM1	PMID: 1378450
SELE	CD44	PMC4571854
SELE	GLG1	PMID: 11404363

The ligands are IPF-DEgenes. The evidence for each ligand–receptor pair is indicated as described in CellChatDB. *ACKR1* atypical chemokine receptor 1, *ACKR3* atypical chemokine receptor 3, *CCL18* C–C motif chemokine ligand 18, *CD44* cluster of differentiation 44, *CEACAM1* CEA cell adhesion molecule 1, *CXCL9* C–X–C motif chemokine ligand 9, *CXCL10* C–X–C motif chemokine ligand 10, *CXCL11* C–X–C motif chemokine ligand 11, *CXCR3* C–X–C motif chemokine receptor 3, *GLG1* Golgi glycoprotein 1, *IFNG* interferon gamma, *IFNGR1* interferon gamma receptor 1, *IFNGR2* interferon gamma receptor 2, *IL6* interleukin 6, *IL6R* interleukin 6 receptor, *IL6ST* interleukin 6 cytokine family signal transducer, *SELE* selectin E.

Step 2: To identify the receptors for the ligands in 1 (via CellChatDB as described in Fig. 4).

For the seven ligands identified in Step 1 above, CellChatDB identified eight receptors (corresponding to 10 genes) (atypical chemokine receptor 1: ACKR1, CXCR3, atypical chemokine receptor 3: ACKR3, interleukin 6 receptor/interleukin 6 cytokine family signal transducer: IL6R/IL6ST, interferon gamma receptor 1/interferon gamma receptor 2: IFNGR1/IFNGR2, CEA cell adhesion molecule 1: CEACAM1, cluster of differentiation 44: CD44, Golgi glycoprotein 1: GLG1), forming 13 ligand–receptor pairs (CCL18-ACKR1, CXCL9-ACKR1, CXCL10-ACKR1, CXCL11-ACKR1, CXCL9-CXCR3, CXCL10-CXCR3, CXCL11-CXCR3, CXCL11-ACKR3, IL6-IL6R/IL6ST, IFNG-IFNGR1/IFNGR2, SELE-CEACAM1, SELE-CD44, SELE-GLG1) (Table 1).

Step 3: To identify the receptors in the lung DEgenes (via CellChatDB as described in Fig. 4).

The CellChatDB analysis identified nine receptors (C–X–C motif chemokine receptor 3: CXCR3, C–X–C motif chemokine receptor 5: CXCR5, C–X–C motif chemokine receptor 6: CXCR6, bradykinin receptor B1: BDKRB1, cholinergic receptor nicotinic alpha 1 subunit: CHRNA1, transmembrane and immunoglobulin domain containing 3: TMIGD3, desmocollin 3: DSC3, programmed cell death 1: PDCD1, SELE) (Table 2).

**Table 2:** The receptors encoded by the IPF-DEgenes and their ligands.

Ligand	Receptor (IPF-DEgene)	Evidence
PF4V1	CXCR3	KEGG: hsa04060
CXCL9	CXCR3	KEGG: hsa04060
CXCL10	CXCR3	KEGG: hsa04060
CXCL11	CXCR3	KEGG: hsa04060
CXCL13	CXCR3	KEGG: hsa04060
PF4	CXCR3	KEGG: hsa04060
CXCL13	CXCR5	KEGG: hsa04060
CXCL16	CXCR6	KEGG: hsa04060
KNG1	BDKRB1	KEGG: hsa04080
SLURP1	CHRNA1	KEGG: hsa04080
SLURP2	CHRNA1	KEGG: hsa04080
ENTPD1	TMIGD3	PMID: 21677139
DSG1	DSC3	PMID: 27298358
DSG2	DSC3	PMID: 27298358
CD274	PDCD1	PMID: 23954143
PDCD1LG2	PDCD1	PMID: 23954143
SELPLG	SELE	PMC4571854

The receptors are IPF-DEgenes. The evidence for each ligand–receptor pair is indicated as described in CellChatDB. *BDKRB1* bradykinin receptor B1, *CD274* cluster of differentiation 274, *CHRNA1* cholinergic receptor nicotinic alpha 1 subunit, *CXCL9* C–X–C motif chemokine ligand 9, *CXCL10* C–X–C motif chemokine ligand 10, *CXCL11* C–X–C motif chemokine ligand 11, *CXCL13* C–X–C motif chemokine ligand 13, *CXCL16* C–X–C motif chemokine ligand 16, *CXCR3* C–X–C motif chemokine receptor 3, *CXCR5* C–X–C motif chemokine receptor 5, *CXCR6* C–X–C motif chemokine receptor 6, *DSG1* desmoglein 1, *DSG2* desmoglein 2, *DSC3* desmocollin 3, *ENTPD1* ectonucleoside triphosphate diphosphohydrolase 1, *KNG1* kininogen 1, *PF4* platelet factor 4, *PF4V1* platelet factor 4 variant 1, *PDCD1* programmed cell death 1, *PDCD1LG2* programmed cell death 1 ligand 2, *SELE* selectin E, *SELPLG* selectin P ligand, *SLURP1* secreted LY6/PLAUR domain containing 1, *SLURP2* secreted LY6/PLAUR domain containing 2, *TMIGD3* transmembrane and immunoglobulin domain containing 3.

Step 4: To identify the ligands for the receptors in 3 (via CellChatDB as described in Fig. 4).

For the nine receptors identified in Step 3 above, CellChatDB identified 16 ligands (platelet factor 4 variant 1: PF4V1, CXCL9, CXCL10, CXCL11, C–X–C motif chemokine ligand 13: CXCL13, platelet factor 4: PF4, C–X–C motif chemokine ligand 16: CXCL16, kininogen 1: KNG1, secreted LY6/PLAUR domain containing 1: SLURP1, secreted LY6/PLAUR domain containing 2: SLURP2, ectonucleoside triphosphate diphosphohydrolase 1: ENTPD1, desmoglein 1: DSG1, desmoglein 2: DSG2, cluster of differentiation 274: CD274, programmed cell death 1 ligand 2: PDCD1LG2, selectin P ligand: SELPLG), forming 17 ligand–receptor pairs (PF4V1–CXCR3, CXCL9–CXCR3, CXCL10–CXCR3, CXCL11–CXCR3, CXCL13–CXCR3, PF4–CXCR3, CXCL13–CXCR5, CXCL16–CXCR6, KNG1–BDKRB1, SLURP1–CHRNA1, SLURP2–CHRNA1, ENTPD1–TMIGD3, DSG1–DSC3, DSG2–DSC3, CD274–PDCD1, PDCD1LG2–PDCD1, SELPLG–SELE) (Table 2).

Step 5: To identify downstream and upstream targets of the ligand–receptor pairs found in Steps 2 and 4 by Kyoto encyclopedia genes and genomes (KEGG) pathway analysis and to select those that are among the 83 latent IPF-features identified by the multi-modal generative toping modeling.

We searched for the downstream and upstream signaling targets for these ligand–receptor pairs by the KEGG-mining (see “Methods” section). For the 13 ligand (lung)–receptor (non-lung) pairs (from Step 2, Table 1), we found six such targets in the latent IPF-features (Table 3)—engulfment and cell motility 1 (ELMO1) as the downstream target for CXCL9–CXCR3, CXCL10–CXCR3, and CXCL11–CXCR3 pairs, calcium/calmodulin dependent protein kinase IV (CAMK4) as the downstream target for the IFNG–IFNGR1 and IFNG–IFNGR2 pairs, and phosphoenolpyruvate carboxykinase 1 (PCK1) as the downstream target for the IL6–IL6R pair. In addition, calmodulin 1/calmodulin 2/calmodulin 3 (CALM1/CALM2/CALM3) were identified as upstream targets for the IL6-IL6R pair.

**Table 3:** KEGG pathways of the ligand (IPF-DEgenes)-receptor pairs and their signaling molecules (IPF-features).

Pathway	Ligand (IPF-DEgene)	Receptor	Latent disease-omics feature (IPF-feature)	Position
hsa04062	CXCL9	CXCR3	ELMO1	Downstream
hsa04062	CXCL10	CXCR3	ELMO1	Downstream
hsa04062	CXCL11	CXCR3	ELMO1	Downstream
hsa04380	IFNG	IFNGR1	CAMK4	Downstream
hsa04380	IFNG	IFNGR2	CAMK4	Downstream
hsa04151	IL6	IL6R	PCK1	Downstream
hsa05163	IL6	IL6R	CALM1	Upstream
hsa05163	IL6	IL6R	CALM2	Upstream
hsa05163	IL6	IL6R	CALM3	Upstream

The Pathways are from the KEGG pathways (human). The position indicates whether the corresponding latent disease-omics feature is the downstream or the upstream of the ligand–receptor pair in the corresponding KEGG pathway. *CALM1* calmodulin 1, *CALM2* calmodulin 2, *CALM3* calmodulin 3, *CAMK4* calcium/calmodulin dependent protein kinase IV, *CXCL9* C–X–C motif chemokine ligand 9, *CXCL10* C–X–C motif chemokine ligand 10, *CXCR3* C–X–C motif chemokine receptor 3, *ELMO1* engulfment and cell motility 1, *IFNG* interferon gamma, *IFNGR1* interferon gamma receptor 1, *IFNGR2* interferon gamma receptor 2, *IL6* interleukin 6, *IL6R* interleukin 6 receptor, *PCK1* phosphoenolpyruvate carboxykinase 1. KEGG pathways: hsa04062: Chemokine signaling pathway; hsa04380: Osteoclast differentiation; hsa04151: PI3K-Akt signaling pathway; hsa05163: Human cytomegalovirus infection.

For the 17 ligand (non-lung)–receptor (lung) pairs (from Step 4, Table 2), we identified four targets (Table 4), ELMO1 as the downstream target for the PF4V1–CXCR3, CXCL9–CXCR3, CXCL10–CXCR3, CXCL11–CXCR3, CXCL13–CXCR3, CXCL13–CXCR5, PF4–CXCR3, and CXCL16–CXCR6 pairs, CALM1/CALM2/CALM3 as the downstream target for the KNG1–BDKRB1 pair.

**Table 4:** KEGG pathways of the ligand–receptor (IPF-DEgenes) pairs and their signaling molecules (IPF-features).

Pathway	Ligand	Receptor (IPF-DEgenes)	Latent disease-omics feature (IPF-feature)	Position
hsa04062	PF4V1	CXCR3	ELMO1	Downstream
hsa04062	CXCL9	CXCR3	ELMO1	Downstream
hsa04062	CXCL10	CXCR3	ELMO1	Downstream
hsa04062	CXCL11	CXCR3	ELMO1	Downstream
hsa04062	CXCL13	CXCR3	ELMO1	Downstream
hsa04062	CXCL13	CXCR5	ELMO1	Downstream
hsa04062	PF4	CXCR3	ELMO1	Downstream
hsa04062	CXCL16	CXCR6	ELMO1	Downstream
hsa05200	KNG1	BDKRB1	CALM1	Downstream
hsa05200	KNG1	BDKRB1	CALM2	Downstream
hsa05200	KNG1	BDKRB1	CALM3	Downstream

The Pathways are from the KEGG pathways (human). The position indicates whether the corresponding latent disease-omics feature is the downstream or the upstream of the ligand–receptor pair in the corresponding KEGG pathway. *BDKRB1* bradykinin receptor B1, *CALM1* calmodulin 1, *CALM2* calmodulin 2, *CALM3* calmodulin 3, *CXCL9* C–X–C motif chemokine ligand 9, *CXCL10* C–X–C motif chemokine ligand 10, *CXCL11* C–X–C motif chemokine ligand 11, *CXCL13* C–X–C motif chemokine ligand 13, *CXCL16* C–X–C motif chemokine ligand 16, *CXCR3* C–X–C motif chemokine receptor 3, *CXCR5* C–X–C motif chemokine receptor 5, *CXCR6* C–X–C motif chemokine receptor 6, *ELMO1* engulfment and cell motility 1, *KNG1* kininogen 1, *PF4* platelet factor 4, *PF4V1* platelet factor 4 variant 1. KEGG pathways: hsa04062: Chemokine signaling pathway; hsa05200: Pathways in cancer.

Step 6: To identify cell-types where the ligands-receptors and their downstream and upstream singling targets found in Steps 1–5.

Our aim is to identify the inter-organ mechanism of IPF. The multi-modal generative topic-model found a possible involvement of the liver in this mechanism (Fig. 2). Hence, the ligand–receptor pair(s) that bridge the lung (the primary organ of IPF pathology) and the liver could be such a mechanism. Furthermore, to fulfill this mechanism, the expression of the non-lung component of the ligand–receptor pair should be enriched in the liver.

**Figure 5:**
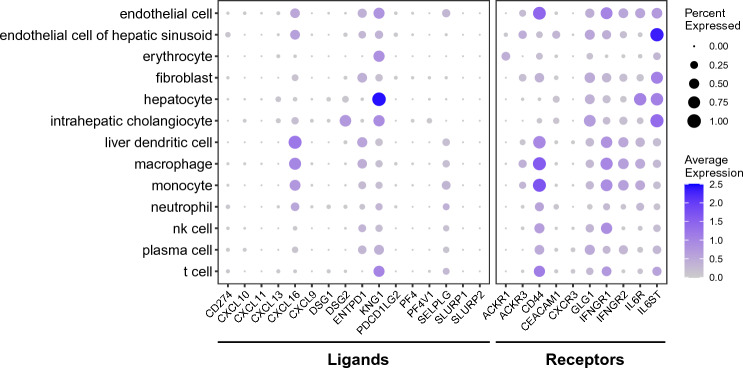
The hepatic expression of the ligands and receptors for the IPF pulmonary receptors and ligands. The level of each ligand and receptor in each cell-type in the liver is shown as dot. The size and the heat-intensity represent the ratio of cells expressing the gene in each cell-type cluster and the mean expression level of log-transformed counts [i.e., log(1 + count per 10,000)], respectively, as shown on the right side of the panel. The raw data are available as Supplementary Table S5. nk cell: natural killer cell.

On the basis of this rationale, we examined the expression patterns of the non-lung components of the ligand–receptor pairs in the liver (Fig. 5, Supplementary Table S5). The analysis of the multi-organ human single-cell RNA sequencing (scRNA-seq) database, *Tabula Sapiens*, identified two pairs, KNG1 (the liver)–BDKRB1 (the lung) and IL6 (the lung)–IL6R/IL6ST (the liver), that could establish the lung-liver inter-organ mechanism. The expression of KNG1, the ligand for BDKRB1, is most enriched in hepatocytes, with lesser expression in the endothelial cells, fibroblasts, intrahepatic cholangiocytes, and T cells. The expression of IL6R/IL6ST, the receptor complex for IL6, is enriched in the endothelial cells of the hepatic sinusoid, intrahepatic cholangiocytes, and hepatocytes.

**Figure 6:**
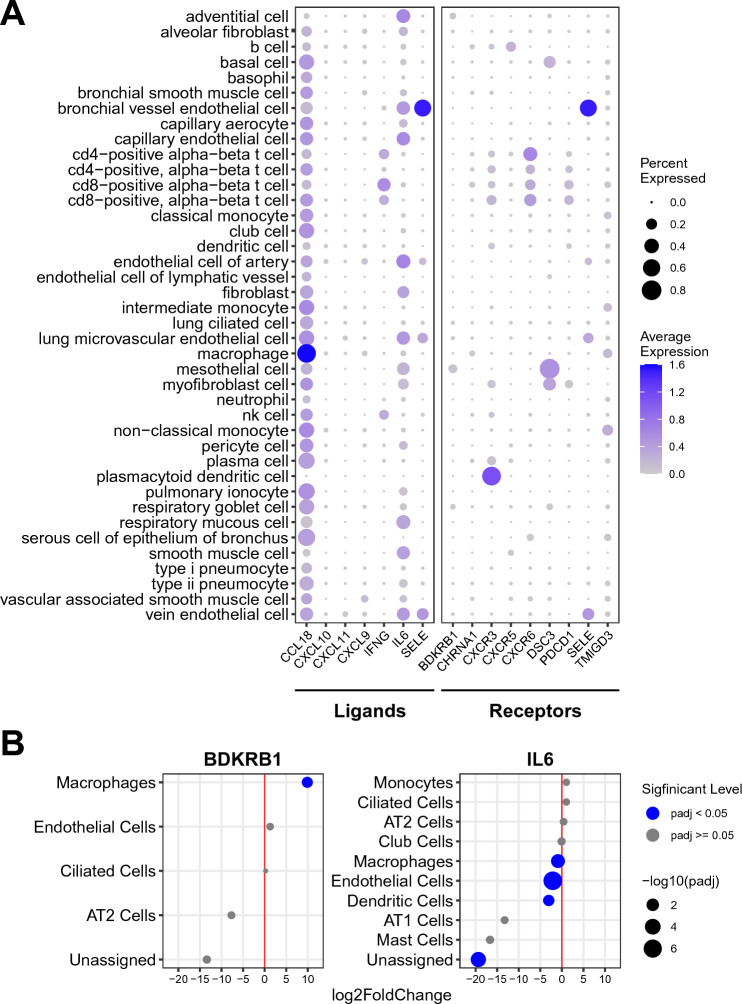
The expression of IL6 and BDKRB1 in the lung. (**A**) The level of each ligand and receptor (including IL6 and BDKRB1) in each cell-type in the lung of the healthy subjects (*Tabula Sapiens*) is shown as dot. The size and the heat-intensity represent the ratio of cells expressing the gene in each cell-type cluster and the mean expression level of log-transformed counts [i.e., log(1 + count per 10,000)], respectively, as shown on the right side of the panel. The raw data are available as Supplementary Table S6. nk cell: natural killer cell. (**B**) The differential expression of IL6 and BDKRB1 in each cell-type in the IPF-lung is shown as dot. The cell-types are indicated on the left. The differential expression of IPF vs. non-IPF is indicated as log_2_fold change (“log2FoldChange”). The dot size indicates the statistical significance of the differential expression as − log_10_p-adj (“− log10padj”)—the larger size indicating more significant (i.e., less padj values). The blue and gray colors indicate padj $<$ 0.05 and padj $\ge$ 0.05, respectively. The raw data are available as Supplementary Table S7. *padj* adjusted p-value, *AT1 cells* alveolar type I cells, *AT2 cells* alveolar type II cells.

Next, we examined the expression patterns of their partner components in the lung (Fig. 6, Supplementary Tables S6 and S7), which were originally found in the 112 IPF-lung DEgenes. The scRNA-seq analysis of IL6, the ligand for the IL6R/IL6ST receptor complex, in the lung using the *Tabula Sapiens* was performed. The result shows that the enrichment of IL6 expression in adventitial cells, endothelial cells, fibroblasts, mesothelial cells, respiratory mucous cells, and smooth muscle cells (Fig. 6A, Supplementary Table S6). We also examined whether the expression pattern of IL6 in the lung is altered in the IPF patients (Fig. 6B, Supplementary Table S7). The result identified over twofold downregulation of the IL6 expression in endothelial cells and dendritic cells in the IPF lung. In addition, in the lung macrophage, its nearly twofold downregulation was also found. While the expression of BDKRB1 is detected more ubiquitously in the lung (Fig. 6A, Supplementary Table S6), it is nearly 1000-fold upregulated in the macrophages of the IPF lung, as compared to those of the healthy lung (Fig. 6B, Supplementary Table S7).

**Figure 7:**
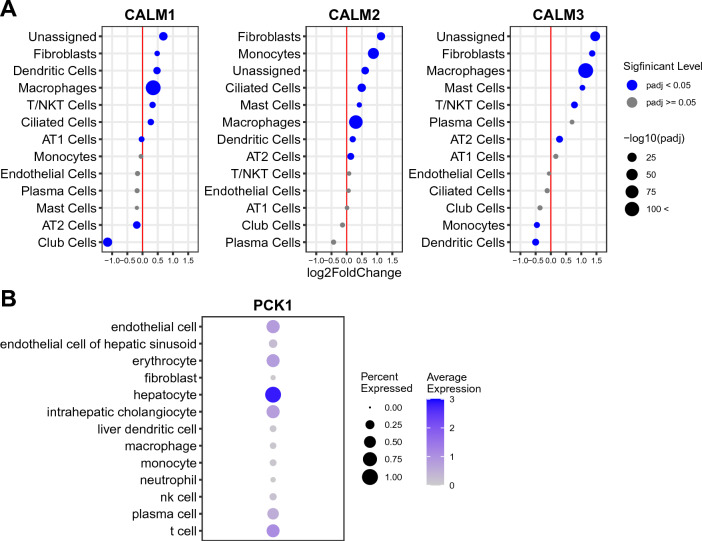
The expression of the signaling targets in the liver and the lung. (**A**) The differential expression of CALM1/CALM2/CALM3 in each cell-type in the IPF-lung is shown as dot. The cell-types are indicated on the left. The differential expression of IPF vs. non-IPF is indicated as log_2_fold change (“log2FoldChange”). The dot size indicates the statistical significance of the differential expression as − log_10_p-adj (“− log10padj”)—the larger size indicating more significant (i.e., less padj values). The blue and gray colors indicate padj $<$ 0.05 and padj $\ge$ 0.05, respectively. The raw data are available as Supplementary Table S7. padj: adjusted p-value; AT1 cells: alveolar type I cells; AT2 cells: alveolar type II cells. T/NKT cells: T/natural killer T cells. (**B**) The level of PCK1 in each cell-type in the liver is shown as dot. The size and the heat-intensity represent the ratio of cells expressing the gene in each cell-type cluster and the mean expression level of log-transformed counts [i.e., log(1 + count per 10,000)], respectively, as shown on the right side of the panel. The raw data are available as Supplementary Table S5. *nk cell* natural killer cell.

We also examined the expression patterns of their downstream and upstream signaling targets (Fig. 7). The most significant upregulation of CALM1/CALM2/CALM3, the downstream targets of the KNG1–BDKRB1 signaling and the upstream targets of the IL6–IL6R/IL6ST signaling, was detected in macrophages in the IPF lung (Fig. 7A, Supplementary Table S6). Lesser but statistically significant upregulation for one or more of these targets was also found in fibroblasts, dendritic cells, T/natural killer T (T/NKT) cells, ciliated cells, monocytes, mast cells, and alveolar type II cells (AT2) cells. Small but statistically significant downregulation was observed for CALM1 in alveolar type I cells (AT1) cells, AT2 cells, and club cells. Such downregulation was also detected for CALM3 in monocytes and dendritic cells.

The expression pattern of PCK1, the downstream target of IL6-IL6R/IL6ST signaling, was examined in the liver (Fig. 7B, Supplementary Table S6). The result shows its highest expression in hepatocytes. Its less abundant expression is detected in endothelial cells, erythrocytes, intrahepatic cholangiocytes, plasma cells, and T cells.

Step 7: To construct the inter-organ map on the basis of 1–6 results.

**Figure 8:**
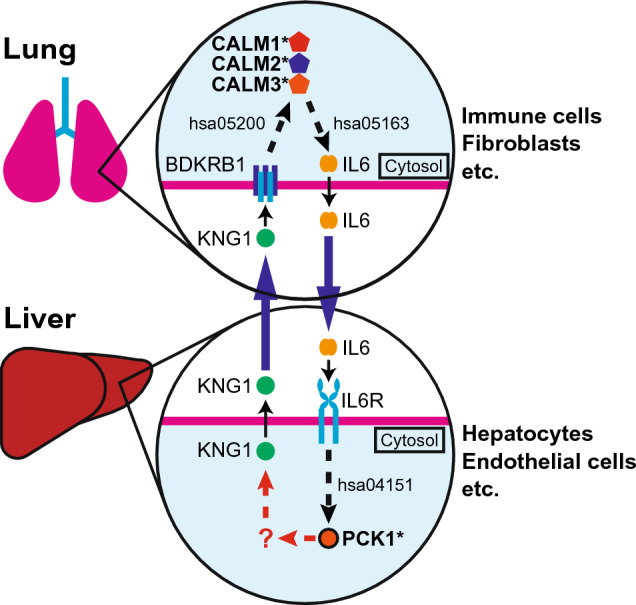
The predicted inter-organ mechanism of IPF. The solid arrows indicate the direct ligand–receptor interactions. The pathway connection (edge) is shown as dashed-arrows indicating the presence of one or more nodes (proteins: ligands, receptors, signaling targets) in between. The corresponding KEGG human pathways for each edge are indicated as hsa numbers. “?” indicates the lack of KEGG pathway connecting the nodes. *BDKRB1* bradykinin receptor B1, *CALM1/2/3* calmodulin 1/2/3, *IL6* interleukin 6, *IL6R* interleukin 6 receptor, *KNG1* kininogen 1, *PCK1* phosphoenolpyruvate carboxykinase 1. KEGG pathways: hsa04151: PI3K-Akt signaling pathway; hsa05163: Human cytomegalovirus infection pathway; hsa05200: Pathways in cancer.

We put together the results obtained through Steps 1–6 and generated a landscape representing an inter-organ mechanism of IPF (Fig. 8). The logic is as follows: KNG1, expressed in the hepatic cells (Fig. 5), is the systemic ligand for its receptor, BDKRB1 (Table 2). BDKRB1 is also one of the 112 IPF-DEgenes expressed in the pulmonary cells (Table 1, Fig. 6B, Supplementary Table S1). Hence, the hepatic KNG1 directly interacts with pulmonary BDKRB1 across these organs (Fig. 8). CALM1/CALM2/CALM3, the latent IPF-features (Supplementary Table S1) are the known downstream targets of KNG1 (ligand)—BDKRB1 (receptor) signaling (KEGG: hsa05200, Pathways in cancer) (Table 4). In addition, CALM1/CALM2/CALM3 are also known upstream signaling components of the IL6 signaling (KEGG: hsa05163, Human cytomegalovirus infection pathway) (Table 3). CALM1/CALM2/CALM3 are expressed in the pulmonary cells and their expression is upregulated in the pulmonary macrophages and fibroblasts, etc. of the IPF lung (Fig. 7A). IL6 is one of the IPF-DEgenes (Fig. 6B, Supplementary Table S4) and is a systemic ligand for its receptor, IL6R/IL6ST (Table 1). IL6R/IL6ST complex is expressed in hepatic cells (Fig. 5). Hence, the signal from the liver is transduced to the lung via KNG1 (ligand)–BDKRB1 (receptor) interaction across these organs via CALM1/CALM2/CALM3 to the IL6 signal in the lung (Fig. 8). This pulmonary IL6 signal is transduced back to the liver via the IL6 (ligand)–IL6R/IL6ST (receptor) interaction in the liver (Fig. 8). PCK1, one of the IPF disease-omics features, is a known signaling molecule for the IL6 (ligand)–IL6R/IL6ST (receptor) interaction (KEGG: hsa04151, PI3K-Akt signaling pathway), and it is expressed in the hepatic cells (Fig. 7). Hence the IL6 signal from the IPF-lung is transduced in the liver via PCK1 signaling molecule (Fig. 8). With this logic, the mechanism described in Fig. 8 is generated. In this mechanism, the liver-derived KNG1 activates the CALM1/CALM2/CALM3 signaling pathway via BDKRB1 in the lung. This signal amplifies the expression and/or secretion of IL6 from the lung. The systemic IL6 activates the PCK1 signaling pathway via IL6R/IL6ST in the liver. This feedforward and feedback mechanism across the liver and the lung triggers and/or exacerbates IPF pathogenesis.

## Discussion

While the results shown in this study are computational, there are mounting pre-clinical and clinical evidence supporting our findings. They are as follows (below).

### The latent relatedness of IPF to non-pulmonary diseases

By applying the multi-modal generative topic modeling to the multi-modal disease-omics data of 6,955 human diseases, we identified molecular and genetic relatedness of IPF to non-respiratory diseases such as various types of neoplasm, autoimmune disorders, diabetes, Alzheimer’s disease, rheumatoid arthritis, obesity, cardiovascular diseases (atherosclerosis, arteriosclerosis, hypertensive disease, etc.), systemic lupus erythematosus, and multiple sclerosis (Fig. 3). A possible similarity of IPF to lung cancer is discussed in an editorial article^30^. In this article, rhodopsin guanine nucleotide exchange factors (RhoGEF) mediated epithelial cell transformation (ECT) 2 of AT2 cells in the lung could be a common mechanism between IPF and the lung cancer. Our topic modeling study shows the relatedness of IPF to diverse types of cancer (Fig. 3). As AT2 is a relatively specific resident cell-type in the lung, it is unlikely that the same mechanism is the basis of the relatedness of IPF to other non-lung cancers. However, a possibility of the ECT of other types of epithelial cells in non-lung tissues remains, which could explain the relatedness of IPF to other types of cancer as found in our study.

The relatedness of IPF to diabetes is another finding worth discussion. There are several clinical studies including clinical meta analyses suggesting an association between IPF and diabetes^31–34^. Our computational study also indicates molecular relatedness of IPF to diabetes (Fig. 3). Moreover, other IPF-related diseases found in this study such as Alzheimer’s disease, obesity, cardiovascular diseases, systemic lupus erythematosus are linked to diabetes^35–39^. Furthermore, the two signaling nodes in the inter-organ mechanism of IPF proposed in our study (Fig. 8), CALM1/CALM2/CALM3 (in the lung) and PCK1 (in the liver), are both molecularly linked to diabetes (Supplementary Table S1). Taken together, IPF may share the same molecular underpinnings with diabetes and other related diseases (i.e., Alzheimer’s disease, obesity, cardiovascular diseases, systemic lupus erythematosus).

### The inter-organ mechanism of IPF

Our generative computational approach predicts a molecular crosstalk mechanism between the lung and the liver for IPF (Fig. 8). The possibility of the lung-liver interaction in IPF is further supported by a clinical observation of liver fibrosis in some IPF patients^40^.

In the lung-liver interaction mechanism that we found, two secreted systemic factors, KNG1 and IL6, bridge the liver-lung crosstalk. Hence, based on this mechanism, the interference of KNG1–BDKRB1(a receptor for KNG1) and/or IL6-IL6R (a receptor for IL6) interactions could bring a therapeutic benefit to IPF. In this regard, it is worth noting that the blocking IL-6 is shown to attenuate pulmonary fibrosis in mice^41^.

In the same study, it is also shown that IPF patients exhibit the increased level of soluble interleukin 6 receptor subunit alpha (sIL-6Rα) in their lung tissues. However, the proposed therapeutic mechanism is the blocking of the intra-pulmonary interactions of sIL-6Rα and IL6. Furthermore, possible roles of interleukins in the pathogenesis of pulmonary fibrosis including IPF are recently discussed^42^. In these other studies, the IL6 inhibitory effects in IPF patients are discussed only in the context of heterologous cell–cell crosstalk within the lung tissue. However, as the IL6R is also present in hepatocytes, hepatic endothelial cells, and intrahepatic cholangiocytes (Fig. 5), such IL6–IL6R inhibitory effect could also occur in the liver. Hence, when the therapeutic inhibition of the IL6–IL6R interaction is effective, it is important to consider a possibility that such effects are also through the inhibition of this ligand–receptor interaction outside the lung tissue such as within the liver tissue.

In our inter-organ mechanism, we also propose that the calmodulin pathway (CALM1/CALM2/CALM3) is activated by the liver-derived KNG1 interaction with the lung BDKRB1, which then induces the IL6 pathway (Fig. 8). It is shown that a calmodulin inhibitor, trifluoperazine, exhibits an anti-inflammatory effect in a bleomycin-induced pulmonary fibrosis animal model^43^. This pre-clinical evidence supports our proposed mechanistic model.

The other signaling node in our inter-organ mechanism is PCK1 (Fig. 8). In our model, PCK1 pathway is activated by the IL6-IL6R interaction in the liver, which is feeds back to the lung pathogenesis of IPF via KNG1-BDKRB1 pathway. Recently, nintedanib, one of the two FDA-approved IPF therapeutics, is shown to attenuate experimental colitis via inhibiting the PCK1 pathway^44^. This study suggests that a part of the therapeutic effect of nintedanib on IPF is via the inhibition of the PCK1 pathway.

There are two pending questions in the proposed inter-organ mechanism of IPF. The ligands, receptors, and their signaling targets in this model are co-expressed in multiple cell types in their corresponding organs (Fig. 8). Hence, it remains unknown whether the KNG1–BDKRB1 and IL6–IL6R/IL6ST pathways function within the same cell-type or they interact in *trans* across different cell-types within the same organ.

Another question is whether the IL6–IL6R/IL6ST signal feeds back to KNG1 via PCK1 (Fig. 8). While the signaling of IL6–IL6R/IL6ST to PCK1 is established (hsa04151 KEGG pathway, PI3K-Akt signaling pathway in human), the link of PCK1 to KNG1 remains unknown. Upon the experimental validation of this link, the model becomes a closed feedforward and feedback “loop” across the liver and the lung.

These questions remain for the future studies and their results provide more detailed mechanistic description of IPF. Furthermore, they facilitate the designing of first-in-class therapeutic and/or diagnostic strategies for IPF.

In this study, we exploited a growing body of multi-modal disease-omics data and a generative computational power to predict an inter-organ mechanism of IPF with the molecular and cellular resolution. Furthermore, our retrospective reference-mining found multiple experimental and clinical evidence in support of the predicted mechanism as described above. Our proposed mechanism is detailed enough, providing a unique opportunity to design hypothesis-driven pre-clinical experiments and/or clinical studies to discover and evaluate first-in-class therapeutic and diagnostic targets for IPF. In addition, our study and results illustrate a computational framework to generate experimentally-testable mechanistic models for other diseases where very little mechanism is known.

## Methods

### Multi-modal generative topic modeling

The multi-modal generative topic modeling approach is as previously described^29^. This topic modeling approach exploits the similarities among diseases on the basis of their multi-modal omics features. In this study, we deleted IPF disease-omics data to identify latent IPF-features (see also Fig. 1 and the details in “Result” section).

### Organ- and cell-type expression patterns of the latent IPF-features

The organs and cells where the IPF-features are expressed were identified by organ/cell enrichment analyses using THE HUMAN PROTEIN ATLAS v 21.1^23–25^, as previously described^29^. Briefly, we generated a 2 × 2 contingency table showing the number of the genes of interest that are associated with the target organ(s)/cell(s), and performed chi-square test of independence by using the contingency table.

### Latent relatedness of IPF to other diseases

We identified diseases to which IPF is related as previously described^29^. Briefly, the disease-labels of each latent IPF-feature were identified in the Dataset A (Fig. 1) and the frequency of each disease-label was counted. The disease-labels of the higher-frequency are determined as more related to IPF.

### Lung RNA-seq data from patients

The studies with human subjects and data were approved by the Institutional Review Board of Advanced Telecommunications Research Institute International on behalf of Karydo TherapeutiX, Inc. (Approved Number: HK2101-2101, HK2101-2103, HK2101-2202) and of National Institutes of Biomedical Innovation, Health and Nutrition (Approved Number: 187) and of Kanagawa Cardiovascular and Respiratory Center (Approved Number: KCRC-19-0015). The informed consent was obtained from all subjects. All methods were performed in accordance with the relevant guidelines and regulations. The lung tissues were collected from 299 subjects. They consist of 173 idiopathic interstitial pneumonias (IIPs), 76 hypersensitivity pneumonitis (HP), 26 connective tissue diseases (CTD), 24 others (other interstitial lung diseases). The 173 IIPs are further composed of 95 IPF, 41 unclassifiable interstitial pneumonia (UCIP), 28 idiopathic nonspecific interstitial pneumonia (NSIP), 3 idiopathic pleuroparenchymal fibroelastosis (PPFE), and 6 other IIPs. RNA was purified from each sample and processed for RNA sequencing as follows: The lung tissues were sent to TAKARA BIO INC. (Shiga, Japan) for sequencing. At TAKARA BIO INC., a total RNA was purified using NucleoSpin®RNA according to the provided protocol. Before RNA-sequencing, the total RNA for each specimen was checked for quality using Agilent 2000 TapeStation (Agilent Technologies, Santa Clara, CA, USA). The RNA Integrity Numbers (RINs) for all the specimens, as obtained by TapeStation, passed a score of 6.0 or greater. Upon this quality check, mRNA sequencing was performed using the Illumina Sequencer NovaSeq6000 with paired end reads of 150 bps. Read sequences obtained were mapped to genome sequences. Based on the positional information obtained from the mapping and the gene definition file, the gene units were mapped to the genome sequence. The expression level of each gene and transcript was calculated based on the positional information obtained by mapping and the gene definition file and an annotation information was added. The differentially expressed genes (DEgenes) in the lung tissues of IPF vs. all the other pulmonary diseases (UCIP, NSIP, PPFE, other IIPs (labeled as “IIP” in the raw count data), HP, CTD, other interstitial lung diseases (labeled as “Others” in the raw count data) were detected by using an R package, *DESeq2*^45^, with the default parameter settings. The DEgenes were defined as the genes whose absolute values of log2FoldChange are $\ge 1$ and also adjusted p-values are $<0.05$. These IPF-DEgenes are referred to as “IPF-DEgenes” in this paper.

### Identification of ligand–receptor pairs

From the IPF-DEgenes, those encoding ligand proteins or receptor proteins were identified using a human ligand–receptor combination database, CellChatDB^46^. Furthermore, we identified their corresponding receptor and ligand partners using the same database.

### Generation of an inter-organ map of IPF

The overall design of the approach is described in Fig. 4 (see the “Results” section for its narrative description). The gene expression analyses in the organs and cells were performed as follows:

The single-cell gene expression across multiple healthy organs and that of the lung tissues of IPF patients were determined using two publicly available human single-cell RNA sequencing (scRNA-seq) datasets: (1) *Tabula Sapiens*, which is a database of multiple healthy tissues^22^, and (2) GSE122960, which is a scRNA-seq dataset of the lung tissues of IPF patients/healthy donors^47^. For the *Tabula Sapiens*, the raw count data and the cell type annotation table were extracted using a Python package, *scanpy*^48^, from the h5ad-formatted data at their FigShare deposit^49^. For the GSE122960, the raw count data were obtained from the Gene Expression Omnibus (GEO) deposit. However, the cell type annotation table for GSE122960 was not available. Therefore, we reproduced the cell type annotation from the downloaded count data using publicly available R program codes (https://github.com/NUPulmonary/Reyfman2018/tree/master)^47^, which accompany the GSE122960 study. These codes were run in the *Seurat* package (v2.3.4)^50^ as follows: (1) Cell quality control filtration by the number of genes and the percent of mitochondrial RNA; (2) Log-normalization with the scale factor 10,000; (3) Dection of highly variable genes; (4) Scaling for all genes; (5) Principal component analysis (PCA) and the elbow plot to determine the PCs used for the downstream analyses; (6) Cell clustering and the cell type determination by the marker genes. The parameter settings at each step and the cell type annotation were performed according to the obtained codes per each sample data. The DEgenes in each cell type of the lung derived from the IPF patients were determined by comparing the scRNA-seq data of the lung tissues derived from the IPF patients and the healthy-donors by *DESeq2*^45^ following the developers’ recommendations for single-cell analysis^51^. Briefly, we first set size-factors by ‘computeSumFactors()’ in the *scran* package^52^. The DESeq2 was performed by using the likelihood ratio test as significance testing, where we set the ‘DESeq()’ arguments to the following values: test = ‘LRT’, useT = TRUE, minmu = 1e−6, minReplicateForReplace = Inf. The genes were evaluated by the statistical significance level at 0.05 in adjusted p-value.

The KEGG-mining (steps 5 and 6 in the flow) was conducted as follows:

The KEGG-mining was performed to identify downstream and upstream targets of the ligand–receptor pairs and to determine which of the targets are the IPF-features. First, the gene symbols were converted into KEGG IDs, by first to Entrez IDs using the R function ‘bitr()’ of the R package *clusterProfiler*^53^, and then to KEGG IDs from the Entrez IDs using KEGG API^54^. Next the KEGG pathways containing these KEGG IDs were extracted and their directed graphs were constructed using KGML^55^. In the graphs, each node was the attribute ‘name’ of the tag <entry>, and each edge started at the node corresponding to the attribute ‘entry1’ of the tag <relation> and ended at the node corresponding to the attribute ‘entry2’ of the same tag <relation>. Using these graphs, we identified the direct and indirect connections between the ligands/receptor and the latent IPF-features.

### Ethics approval and consent to participate

The studies with human subjects and data were approved by the Institutional Review Board of Advanced Telecommunications Research Institute International on behalf of Karydo TherapeutiX, Inc. (Approved Number: HK2101-2101, HK2101-2103, HK2101-2202) and of National Institutes of Biomedical Innovation, Health and Nutrition (Approved Number: 187) and of Kanagawa Cardiovascular and Respiratory Center (Approved Number: KCRC-19-0015).

### Supplementary Information

Supplementary Information 1.

Supplementary Table S1.

Supplementary Table S2.

Supplementary Table S3.

Supplementary Table S4.

Supplementary Table S5.

Supplementary Table S6.

Supplementary Table S7.

## Data Availability

The datasets generated and/or analyzed during the current study are available in GEO, GSE122960; figshare repository, https://figshare.com/projects/Tabula_Sapiens/100973; GitHub, https://github.com/skozawa170301ktx/IPF.

## Code availability

All raw data of the results are available as supplementary tables of this paper and they are referenced within the manuscript accordingly. The code reported in this paper is available at: https://github.com/skozawa170301ktx/MultiModalDiseaseModeling.
