# Supplementary material for: Latent inter-organ mechanism of idiopathic pulmonary fibrosis unveiled by a generative computational approach
Source: Sci Rep. 2023 Dec 11;13:21981. doi: 10.1038/s41598-023-49281-0 (PMC10713585; doi:10.1038/s41598-023-49281-0)
Supplement: Supplementary file 1 — Supplementary Information 1. [file 41598_2023_49281_MOESM1_ESM.pdf]

Supplemental information

Supplemental figure

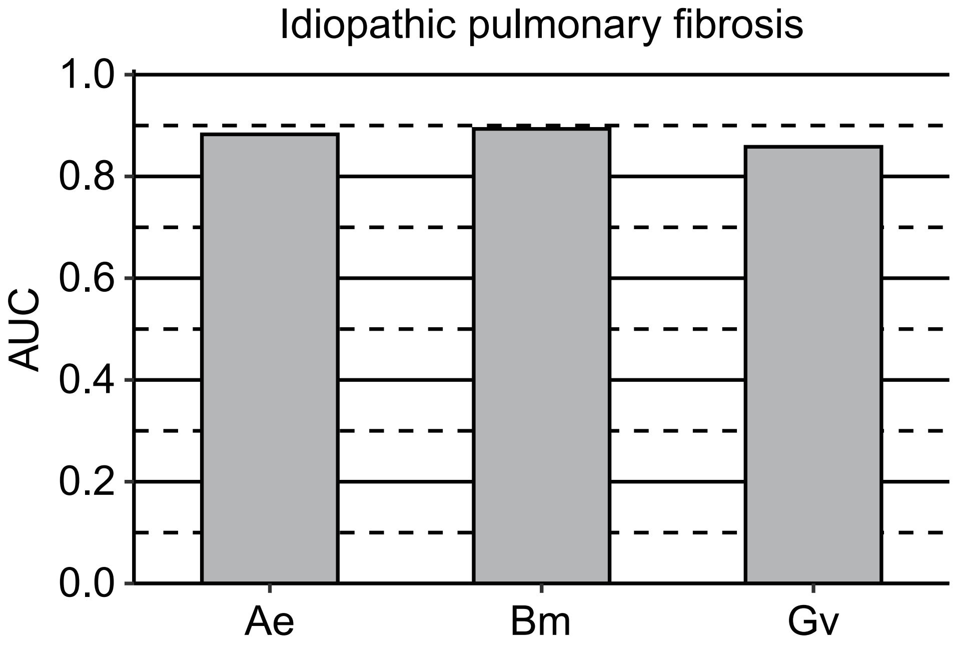

Fig. S1 The AUC scores of each modality for IPF. The AUC scores are calculated for AlteredExpression (Ae), Biomarker (Bm), GeneticVariation (Gv) modalities for IPF and shown as bar graphs. The bars are shown as the AUC scores of 10x leave-one-modality-out cross-validation.

Supplementary tables

Table S1. The list of the 83 latent IPF-features.

Table S2. The organ and cell enrichment of the 83 IPF-features. The raw data for Fig. 2.

Table S3. The disease counts for the 83 IPF-features. The raw data for Fig. 3.

Table S4. Raw data of the DESeq2 analysis. The raw data for the 112 IPF-DEgenes in Fig. 4 are included.

Table S5. The expression of the ligands, the receptors, and PCK1 in each hepatic cell-type (*Tabula Sapiens*). The raw data for Figs. 5&7B.

Table S6. The expression levels of the ligands and receptors in each cell-type in the healthy-lung. The raw data for Fig. 6A.

Table S7. The expression levels of IL6, BDKRB1, and CALM1/CALM2/CALM3 in each cell-type in the IPF-lung. The raw data for Figs. 6B&7A.

List of abbreviations

ACKR1: Atypical chemokine receptor 1

ACKR3: Atypical chemokine receptor 3

Ae: AlteredExpression

AI: Artificial intelligence

AT1 cells: Alveolar type I cells

AT2 cells: Alveolar type II cells

AUC: Area under the curve

BDKRB1: Bradykinin receptor B1

Bm: Biomarker

CALM1: Calmodulin 1

CALM2: Calmodulin 2

CALM3: Calmodulin 3

CAMK4: Calcium/calmodulin dependent protein kinase IV

CCL18: C-C motif chemokine ligand 18

CD44: Cluster of differentiation 44

CD274: Cluster of differentiation 274

CEACAM1: CEA cell adhesion molecule 1

CHRNA1: Cholinergic receptor nicotinic alpha 1 subunit

CTD: Connective tissue diseases

CXCL9: C-X-C motif chemokine ligand 9

CXCL10: C-X-C motif chemokine ligand 10

CXCL11: C-X-C motif chemokine ligand 11

CXCL13: C-X-C motif chemokine ligand 13

CXCL16: C-X-C motif chemokine ligand 16

CXCR3: C-X-C motif chemokine receptor 3

CXCR5: C-X-C motif chemokine receptor 5

CXCR6: C-X-C motif chemokine receptor 6

DEgenes: Differentially expressed genes

DSC3: Desmocollin 3

DSG1: Desmoglein 1

DSG2: Desmoglein 2

ECT: Epithelial cell transformation

ELMO1: Engulfment and cell motility 1

ENTPD1: Ectonucleoside triphosphate diphosphohydrolase 1

FDA: Food and Drug Administration

GEO: Gene Expression Omnibus

GLG1: Golgi glycoprotein 1

Gv: GeneticVariation

HP: Hypersensitivity pneumonitis

hsa: *Homo sapiens*

ID: Identification

IFNG: Interferon gamma

IFNGR1: Interferon gamma receptor 1

IFNGR2: Interferon gamma receptor 2

IIP: Idiopathic interstitial pneumonias

IL6: Interleukin 6

IL6R: Interleukin 6 receptor

IL6Rα: Interleukin 6 receptor subunit α

IL6ST: Interleukin 6 cytokine family signal transducer

IPF: Idiopathic pulmonary fibrosis

KEGG: Kyoto Encyclopedia of Genes and Genomes

KGML: KEGG Markup Language

KNG1: Kininogen 1

mRNA: messenger RNA (Ribonucleic acid)

nk cell: Natural Killer cell

NSIP: Nonspecific interstitial pneumonia

padj: Adjusted p-value

PCK1: Phosphoenolpyruvate carboxykinase 1

PDCD1: Programmed cell death 1

PDCD1LG2: Programmed cell death 1 ligand 2

PF4: Platelet factor 4

PF4V1: Platelet factor 4 variant 1

PMC: PubMed Central

PMID: PubMed ID

PPFE: Pleuroparenchymal fibroelastosis

RhoGEF: Rho (Rhodopsin) guanine nucleotide exchange factors

RINs: RNA integrity Numbers

RNA: Ribonucleic acid

RNA-seq: RNA sequencing

scRNA-seq: single-cell RNA sequencing

SELE: Selectin E

SELPLG: Selectin P ligand

sIL-6Rα: Soluble interleukin 6 receptor subunit α

SLURP1: Secreted LY6/PLAUR domain containing 1

SLURP2: Secreted LY6/PLAUR domain containing 2

TGF-beta: Transforming growth factor-beta

T/NKT cells: T/Natural Killer T cells

TMIGD3: Transmembrane and immunoglobulin domain containing 3

UCIP: Unclassifiable interstitial pneumonia
